# Forgotten Variables and Incarcerated Males

**DOI:** 10.1093/geroni/igaa057.1993

**Published:** 2020-12-16

**Authors:** George Randall, Alex Bishop

**Affiliations:** 1 Sam Houston State University, Huntsville, Texas, United States; 2 Oklahoma State University, Stillwater, Oklahoma, United States

## Abstract

Religiosity tends to mitigate mental health challenges for incarcerated males. Further, negative life events experienced during childhood tend to exacerbate young adult challenges, resulting in incarceration. The current study, based on Koenig (2015) and the Developmental Adaptation Model (DAM; Martin & Martin, 2002), used self-report data from 261 older male inmates, ages 45-82 (M=57.59; SD =8.41) to test a path analytic model regressing religiosity (public religious attendance and private activities) on to known correlates and antecedents. Nested model testing found that the association between self-reported life events experienced as a child and later life religiosity was mediated by forgiveness of self and social provisions, controlling for age, race, and education (IE = -.014; p=.03, one-tail). Distal and proximal assessments influenced religiosity in this sample. Future research will want to explore assessing religiosity and possible therapeutic interventions relative to childhood difficulties and forgiveness of self for incarcerated males.

